# Benefits of Adaptive Sport on Physical and Mental Quality of Life in People with Physical Disabilities: A Meta-Analysis

**DOI:** 10.3390/healthcare11182480

**Published:** 2023-09-07

**Authors:** Eva Isidoro-Cabañas, Francisco Javier Soto-Rodríguez, Francisco Manuel Morales-Rodríguez, José Manuel Pérez-Mármol

**Affiliations:** 1 Distrito Sanitario Metropolitano de Granada, Hospital Universitario Virgen de las Nieves, 18014 Granada, Spain; 2 Departamento de Ciencias de la Rehabilitación, Facultad de Medicina, Universidad de La Frontera, Temuco 4811230, Chile; francisco.soto@ufrontera.cl; 3 Facultad de Ciencias de la Salud, Carrera de Kinesiología, Universidad Autónoma de Chile, Temuco 4780000, Chile; 4 Departmento de Psicología Evolutiva y de la Educación, Facultad de Psicología, Universidad de Granada, 18071 Granada, Spain; fmmorales@ugr.es; 5 Departamento de Fisioterapia, Facultad de Ciencias de la Salud, Universidad de Granada, 18016 Granada, Spain; josemapm@ugr.es; 6 Instituto de Investigación Biosanitaria ibs. GRANADA, 18012 Granada, Spain

**Keywords:** physical disability, young people, adult, adaptive sport, adapted sport

## Abstract

Adaptive sports could produce multiple health benefits in people with physical disabilities. The aim is to evaluate if adaptive sports practice has an influence on physical and mental quality of life. A meta-analysis was performed using electronic databases and other sources. A within- and between-group analysis for physical and mental quality of life was conducted. Standardized mean difference (SMD) was used as a measure of the mean size effect. The statistical heterogeneity, the risk of bias, and the quality of evidence were evaluated. Eight studies met the inclusion criteria and four of them were included in the meta-analysis. In mental quality of life, significant differences were observed in the within-group analysis (SMD = 0.71, *p* < 0.001) and between people practicing adaptive sports and those not engaging in them (SMD = 0.62, *p* = 0.009). In physical quality of life, significant differences were also found between pre- and post-practice of adaptive sports (SMD = 1.03, *p* = 0.007). The engagement in adaptive sports showed a positive impact on the mental quality of life among adults with physical disabilities. However, the positive effect of adaptive sports practice on physical quality of life was shown only in the pre–post-test analysis. Further studies are required to validate the obtained findings.

## 1. Introduction

Physical disability refers to deficiencies, limitations in activities, and restrictions in participation due to dysfunctions in neurological or musculoskeletal systems [1]. Individuals with physical disabilities often have a shorter lifespan than those without such disabilities. Additionally, people with disabilities tend to exhibit lower levels of participation in activities than their non-disabled counterparts [2]. Declerck et al. report less than 20% of people with acquired neurologic damage practice the levels of physical activity recommended by the World Health Organization [3,4]. Another study remarks more than 50% of the spinal cord-injured population is physically inactive [5].

Sport is defined as a physical activity that involves play, games, or competitions, as well as training, adherence to specific rules, and other additional tasks (e.g., warm-up and cool-down activities) typically established by recognized official sports institutions [6,7]. Adaptive sport is conceptualized as a sport that needs the use of specific materials (e.g., wheelchairs designed for participation in athletics track races) or a change in some rules to make it possible for people with disabilities to practice them (e.g., in wheelchair tennis, it is permissible for the ball to bounce twice in the opponent’s court before being returned). Adaptive sport includes different sport modalities specifically created to be practiced by people with disabilities. For instance, boccia is tailored for individuals with physical disabilities [8]. When adaptive sports rules are modified intentionally to use this sport as a therapeutic activity, it becomes a rehabilitation strategy, which has to be supervised by health professionals [9]. The distinction between adaptive sports practice and therapeutic activity is important to analyze the current evidence on the effects of adaptive sports since the literature has mainly focused its attention on using adaptive sports as a therapeutic tool.

Quality of life is regarded as an individual perception of life situation in terms of goals, expectations, and concerns, and it is related to the personal context and the value system of each individual [10,11]. Physical quality of life encompasses an individual’s assessment of various factors, including their ability to manage daily tasks, overall health status, frequency and severity of disease exacerbations, and the impact of received treatments [11]. On the other hand, mental quality of life refers to the assessment that each individual makes of aspects such as his own well-being, his cognitive abilities, and his levels of anxiety, stress, or depression [10,11]. When people suffer from a physical disability and another health condition at the same time, like obesity or mobility problems, functional capacity may be reduced. This fact can, in turn, affect autonomy and quality of life [12]. Individuals with physical disabilities have shown lower mental health outcomes attributed to the challenges arising from the disability and the restricted opportunities for social participation [5].

Regarding the background of the research, adaptive sports practice could be a potential strategy to approach the health conditions present in physical disability, probably generating higher levels of quality of life. Authors such as Aidar et al. found a direct relationship between adaptive sports practice and quality of life in stroke adults [13]. In addition, other researchers such as Ng et al. found that ballroom dancing improves the quality of life in people with multiple sclerosis [14]. However, other authors like Barak et al. found no relationship between the practice of adaptive sports and quality of life [15]. To our knowledge, currently, there are two systematic reviews published on this topic; however, none of them address the following aspects: (i) the isolated examination of the influence of adaptive sport practice on quality of life, (ii) the consideration of the complete diagnostic diversity in the population of adult with physical disability, or (iii) the conduction of meta-analytic analyses [3,12]. One of these reviews [12] included studies that combined adaptive sports and different physical activity interventions; therefore, the isolated effect of adaptive sports practice was not assessed. The other review [3] focused on the population with neurological damage and summarized the influence of adaptive sport on the domains of the International Classification of Functioning, Disability, and Health, without delving into quality of life. In addition, this study lacks a meta-analytic analysis. 

For these reasons, it is necessary to synthesize the existing literature about the influence of the practice of adaptive sports on the quality of life in adults with physical disabilities since available evidence is inconclusive. A meta-analysis would also be crucial to quantify and compare the outcomes from the studies conducted on this topic. In this sense, the aim of this meta-analysis is to investigate the potential impact of adaptive sport practice on both physical and mental quality of life in adults with physical disabilities.

## 2. Methods

### 2.1. Design

The review methodology was prospectively registered with PROSPERO (registration number: CRD42020193791). This study has followed the Preferred Reporting Items for Systematic Reviews and Meta-Analyses (PRISMA) guidelines [16]. The meta-analysis was also performed following general methods for Cochrane reviews.

### 2.2. Search Strategy and Inclusion of Primary Studies

Firstly, it was conducted a search for meta-analyses and systematic or narrative reviews on the study topic in Cochrane Plus, Cochrane Library, the Proquest Platform, and Google Scholar. Reviews on adaptive sports were identified, but no one had the same objective and characteristics as our study.

Then, a database search was performed by two independent investigators in the following databases: Scopus, CINAHL, Web of Science, PubMed, and Medline. The search strategy was built based on the PICO (Population, Intervention, Comparison, Outcome) framework and the use of Booleans operators. Population keywords (e.g., physical disability, young people, adult) were combined with intervention keywords (e.g., adaptive sport, adaptive physical activity, adapted physical activity). The outcome keywords (e.g., quality of life) were not included in the search strategy to avoid missing studies that could be included. Instead, the outcome measures of each study were checked through screening of the available articles. The general search equation was adapted to each database. Filters were used in each database in order to limit the results for publication date and language. As an example, the search strategy used for the Medline database was: (“adapted sport” OR “adaptive sport” OR “adaptive physical activity” OR “adapted physical activity”) AND (young OR adult) AND (“physical disability” OR “physical disabled”). Reference list of reviews and systematic reviews were screened in order to identify other possible eligible studies (backward search). A forward search was also performed based on primary studies found.

The selection criteria for the studies were:

- Study design: studies with quantitative longitudinal design (i.e., randomized controlled trials (RCTs), trials, pre–post design, etc.).
- Study population: adults with physical disability between 18 and 65 years old.
- Interventions: studies evaluating the effects of adaptive sports practice.
- Outcomes: level of physical or mental quality of life.
- Language: English, Spanish or French.
- Publication date: studies published from 2005 to April 2023.

The inclusion of studies with an exclusively longitudinal design was made to enable the meta-analysis results to evaluate the potential relationship between engaging in adaptive sports and possible changes in quality of life. In cases where the rules of an adaptive sport modality were intentionally modified for therapeutic purposes, the authors decided not to include that study in the meta-analysis, considering that the intervention became therapy rather than sport practice. This decision was made with the objective of analyzing only interventions that consisted of engaging in adaptive sports practice.

### 2.3. Study Selection

Primary studies obtained from the different searches were imported into Rayyan [17], software that allows researchers to make decisions independently and remove duplicated items. Following this, two researchers, in parallel and independently, performed a first reading of all the titles and abstracts; if this reading did not indicate that the study should be excluded, it was selected. Subsequently, through the complete reading of the full text of each selected study, compliance with the inclusion criteria was evaluated. Primary studies meeting the inclusion criteria were included and differences of opinion were solved through consensus between two researchers.

### 2.4. Data Extraction and Analysis

A codebook was created *ad hoc* for data extraction in order to record mean, standard deviation, and sample size for each group at pre- and post-evaluations from every primary study. When the published trials provided insufficient data, the authors were contacted to collect the data needed for the meta-analytic analysis. Several pilot trials tested and adjusted the codebook accordingly. Additional outcome data were extracted from each study as well as the characteristics of groups such as duration and frequency. This task was performed simultaneously by two researchers.

With the collected data, it was performed both within-group and between-group analysis for each main outcome measure: physical and mental quality of life. For statistical analysis, Review Manager 5.4.1 software (RevMan) was used. The meta-analytic analyses for the different groups of outcomes were performed by calculating the standardized mean differences (SMD) with the 95% confidence interval. To conduct the within- and between-groups analyses, a random-effect model was chosen by including the pre- and post-intervention means and standard deviations. One included study [15] presents a multi-arm design with three similar interventions based on the practice of adaptive sport and a single control group. To avoid double counts and eliminate unit-of-analysis errors, intervention groups were combined to create a single pair-wise comparison, according to Cochrane Handbook 2022 (version 6.3) (section 23.3.4) [18]. The heterogeneity (using I^2^ index) of included studies were also calculated.

### 2.5. Risk of Bias

The methodological quality of the trials was evaluated using the domain-based assessment recommended in Cochrane Handbook [18]. We used the evaluation of bias tool offered by the software Review Manager 5.4.1. This tool makes possible the evaluation of bias in the same domains purposed by Cochrane and it allows adding other biases that are also relevant. For each trial, the software creates a table where the evaluator must judge the risk of bias for each domain, in accord with “low risk”, “unclear risk” or “high risk”. The evaluator must cite or write the reasons in which the judgment is based.

### 2.6. Quality of Evidence

The quality of the evidence was assessed using the Grading of Recommendations Assessment, Development and Evaluation approach—GRADE [19]. GRADE makes it possible to classify the quality of evidence as “high”, “moderate”, “low” or “Very Low”. This classification is based on the included studies design so that RCTs are considered as high quality of evidence, and observational studies as low-quality evidence. In addition to the design of the studies, the quality of the evidence is determined by evaluating other factors that influence it positively or negatively. The quality of evidence is adversely impacted by several factors, including risk of bias, heterogeneity, indirectness, imprecision, and publication bias. The risk of bias in each included study was assessed by the Cochrane risk of bias tool in Review Manager 5.4.1. The statistical heterogeneity was evaluated using the I^2^ statistic.

## 3. Results

### 3.1. Literature Search

**Figure 1:**
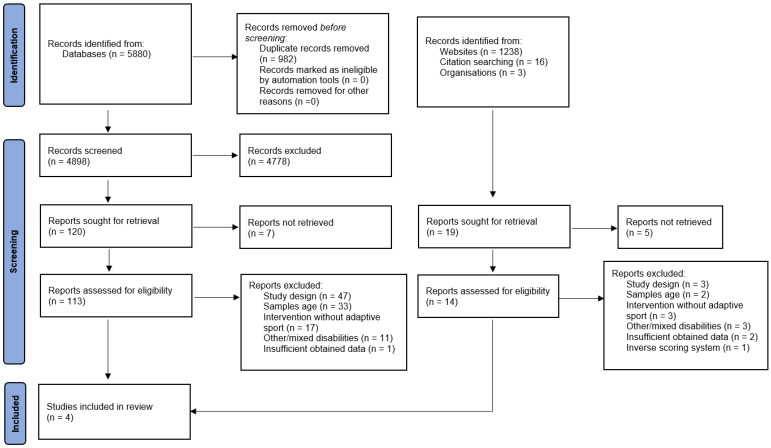
Literature search and screening process—PRISMA flowchart.

A total of 7137 articles were identified through various search strategies. Specifically, 5880 studies were found in the electronic databases search and 1257 studies were found in other electronic sources (grey literature). After removing duplicates, 4898 items were obtained. The main reasons for exclusion were: (i) the study design, (ii) the age of the samples, (iii) interventions not based on adaptive sports, and (iv) samples without physical disability, or mixing physical disability and visual/cognitive disabilities. Figure 1 presents the flow diagram illustrating the process of study selection. After screening titles and abstracts, 127 studies remained. After reading the full texts, only 27 studies evaluated the influence of adaptive sports practice on health aspects in adults with physical disabilities. Of them, eight primary studies focused on the influence of adaptive sports on physical and mental quality of life in adults with physical disabilities. Four of these primary studies were excluded from the meta-analytic analysis because three of them reported insufficient data [20,21,22] and the evaluation instruments (scales) had an inverse scoring system compared to the rest of the included primary studies [23]. Therefore, four primary studies were finally included in the meta-analytic analyses.

### 3.2. Study Characteristics

Of the four included primary studies, one had an RCT design [13], one had a quasi-experimental design [14], one had a pre–post design with three different intervention groups and a control group [15], and one had a single group of repeated measures design with three outcome measures but only two phases, so the intervention group was its own control group [24]. However, the control group could not be included because of missing data at baseline (attrition bias). The total sample of the meta-analysis included 94 individuals between 28 and 62 years old. The samples of the primary studies had physical disabilities such as stroke [13], cerebral palsy, traumatic brain injury, Friedreich ataxia [15], and multiple sclerosis [14,15,24]. Physical and mental quality of life in people with physical disabilities who practice adaptive sports was evaluated by SF-36 [13], WHOQoL-BREF [15], PROMIS-GH [14], and MSQOL-54 [24]. The information about the study characteristics of the studies included in the meta-analysis is gathered and shown in Table 1.

### 3.3. Interventions

Adaptive sports modalities were different in the primary studies. The experimental intervention by Aidar et al. consisted of aquatic activities and swimming sessions of 45–60 min practiced twice a week for 12 weeks. However, the control condition consisted of not receiving any intervention during the trial [13]. The intervention implemented by Ng et al. involved a ballroom dance program consisting of 1 h sessions twice a week over a period of 6 weeks. The control group did not receive any intervention during the study [14]. Intervention by Jackson et al. consisted of kickboxing practice for 1 h, three times per week for 5 weeks [24]. All participants included in the study by Barak et al. received a multidisciplinary rehabilitation program. In addition, the competitive intervention groups trained boccia three times per week for 1.5 h each time and participated in a strength training program twice a week for 1 h. The recreational intervention group participated in two tactics boccia sessions per week too, but not in the competitions and not in a specific training [15]. The available information about the characteristics of the studies included in the meta-analysis is shown in Table 1.

### 3.4. Effects of Adaptive Sport on Physical Quality of Life

**Figure 2:**
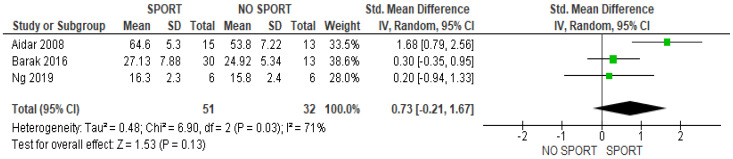
Results of between-group analysis for physical quality of life [13,14,15].

**Figure 3:**
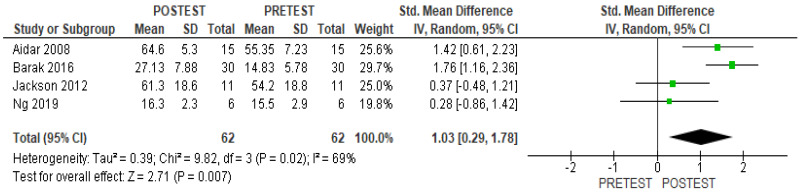
Results of within-group analysis for physical quality of life [13,14,15,24].

The present meta-analysis shows that the practice of adaptive sports did not have effect on physical quality of life when adaptive sports interventions were compared versus a control group (SMD 0.73; 95% CI, −0.21 to 1.67, *p* = 0.13, I^2^ 71%) (Figure 2). However, significant differences were found in the pre–post-test analysis (SMD 1.03; 95% CI, 0.29 to 1.78, *p* = 0.007, I^2^ 69%) (Figure 3).

### 3.5. Effects of Adaptive Sports on Mental Quality of Life

**Figure 4:**
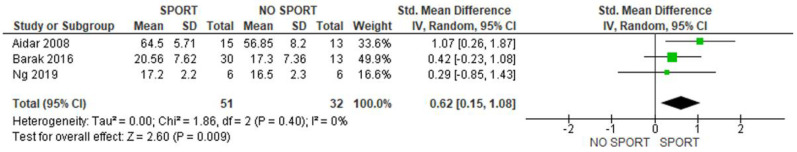
Results of between-group analysis for mental quality of life [13,14,15].

**Figure 5:**
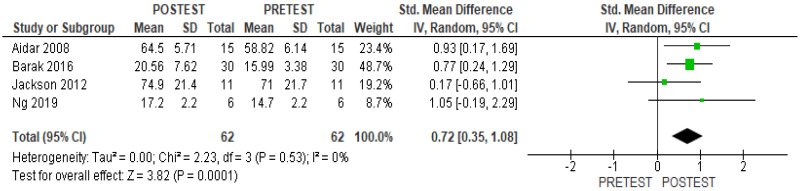
Results of within-group analysis for mental quality of life [13,14,15,24].

Significant differences were found when adaptive sports practice and control groups were compared. This difference was favorable to the group that practiced adaptive sports (SMD 0.62; 95% CI, 0.15 to 1.08, *p* = 0.009, I^2^ 0%) (Figure 4). The pre–post-intervention analysis also showed that adaptive sports practice had a positive effect on the mental quality of life (SMD 0.71; 95% CI, 0.35 to 1.08, *p* = 0.0001, I^2^ 0%) (Figure 5).

### 3.6. Risk of Bias

**Figure 6:**
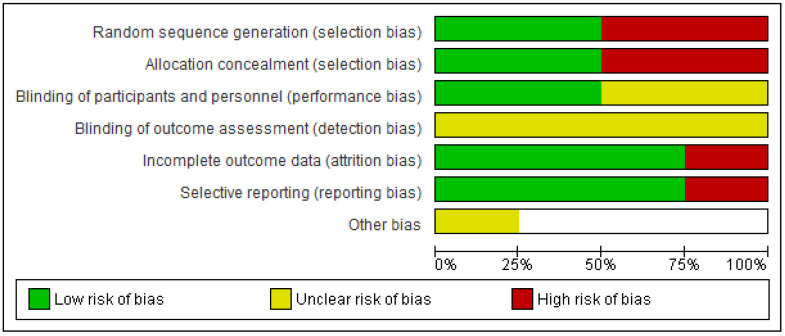
Risk of bias graph: review authors’ judgments about each risk of bias item presented as percentages across all included studies.

**Figure 7:**
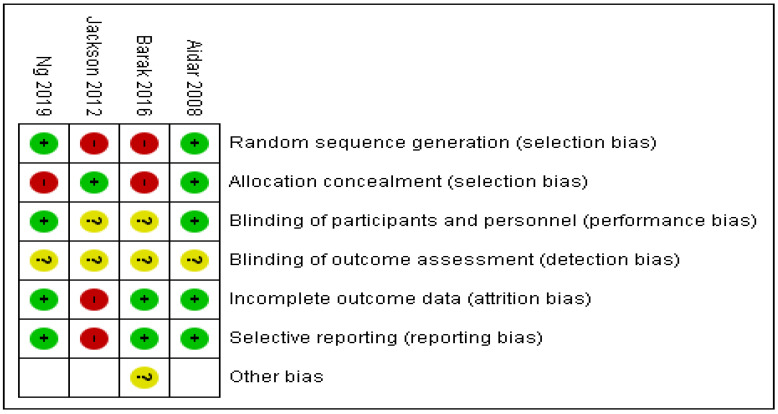
Risk of bias summary: review authors’ judgments about each risk of bias item for each included study [13,14,15,24]. Symbol interpretation: Red (-): high risk of bias; yellow (?): unclear risk of bias; green (+): low risk of bias.

Two of the included primary studies had a high risk of selection bias [13,24] and the other two [14,15] included random allocation. Regarding the “methodological blinding” of the participants and researchers, two primary studies did not collect this information [13,24] so the risk of bias in this aspect is not clear; however, the other two [14,15] showed a low risk. None of the included primary studies reported any information about the detection bias, because the risk of bias is unclearly reported. One of the included primary studies [24] reported a loss of data due to an error in their collection; therefore, it presented a high risk of attrition and reporting bias. On the contrary, the other three included studies presented complete data and they were consistent with the objectives [13,14,15]. In the study by Barak et al. [15] there were other possible biases, which were not possible to assess with precision. On the one hand, great heterogeneity was recognized in the characteristics of the study population. On the other hand, two of the intervention groups performed adaptive sports at a competitive level and the other group at a recreational level. Finally, in terms of the analysis of publication bias, some issues were identified. Despite including all known languages in the search and employing backward and forward search strategies, the limited number of primary studies included in the meta-analysis hinders the meaningful use of funnel plots or other measures to assess this bias effectively. The risk of bias results are depicted in Figure 6 and Figure 7.

### 3.7. Quality of Evidence

The quality of evidence for the influence of the practice of adaptive sports on physical quality of life is low. The included primary studies’ design marked a low quality of evidence. Significant heterogeneity and the high risk of selection bias in three studies and the high risk of attrition bias in one of the included studies decreased the quality of the evidence. A large effect size is considered a factor that improves the quality of the evidence; however, this aspect is not enough to achieve a moderate quality of evidence. Evidence obtained showed differences that were significant in the within-group but not between-group analysis. The practice of adaptive sport revealed a large effect size on the physical quality of life in pre–post-intervention analysis and a moderate effect size in between-groups analysis.

The quality of evidence for the influence of the practice of adaptive sports on mental quality of life is moderate. The included primary studies design marked a low quality of evidence due to the high risk of selection bias in three studies and the high risk of attrition bias in one of the included studies. Evidence obtained showed differences that were significant in both within- and between-group analysis. Furthermore, null heterogeneity could make it increase to a moderate quality of evidence. However, caution should be taken when interpreting the heterogeneity results since the I^2^ value can be influenced by the number of studies included, especially in between-group analyses. The practice of adaptive sports exhibited a moderate effect size on mental quality of life in pre–post-intervention and between-groups analysis.

## 4. Discussion

The main aim of the present study was to evaluate the possible influence of adaptive sports on the physical and mental quality of life of adults with physical disabilities. The practice of adaptive sports revealed a moderate beneficial influence on the physical and mental quality of life of adults with physical disabilities, as evidenced by within-group comparisons. On the other hand, the between-group comparison showed a moderate difference in mental quality of life between adults with physical disabilities who practice adaptive sports and those who do not; however, the post-evaluation scores for physical quality of life were similar. A low quality of evidence was observed for the influence of adaptive sport on physical quality of life and moderate quality of evidence on mental quality of life.

The immediate improvement in physical quality of life can be explained by the protective effect of adaptive sport, since it may reduce the probability of new health events that may deteriorate health [13] and the decrease in fatigue along with the improvement in balance induced by the practice of adaptive sports [14]. On the other hand, the present review found no significant improvement in the physical quality of life of people who practice adaptive sports compared to those who do not. This fact can be explained by the characteristics of the samples and the duration and modalities of the interventions included in the primary studies. For instance, the inclusion of participants with severe physical impairments [15] and the limited duration of interventions, which were restricted to a maximum of twelve weeks [13]. This makes sense since authors such as Laferrier et al. found improvements in the physical quality of life in veterans with disabilities who had participated in adaptive sports programs for more than a year [25]. Nevertheless, authors such as Hutzler et al. neither reported improvements in the physical quality of life in people with severe motor impairment [26].

On the other hand, adaptive sports practice also shows improvement of the mental quality of life of adults with physical disabilities. This finding can be explained by the influence of this type of sports on different cognitive, emotional, and social aspects in this population. A study refers to adaptive sports as a form of leisure once the formal rehabilitation is completed [5]. Various authors point out the effect of adaptive sports in reducing stress and anxiety levels [14,15,27]. These positive effects seem to be greater the longer sports practice lasts [25]. In the same way, adaptive sport has an important role in promoting social and family relationships [28,29]. Hutzler et al. state that adaptive sport significantly improves social competence in people with serious physical impairments [26,30]. Adaptive sport practice offers a means to be socially active and to establish meaningful relationships between equals, reinforcing the emotional aspects [27]. The practice of adaptive sports may favor the creation of an athletic identity [29,31] through the development of self-esteem [28,32]. In line with this athletic identity, adaptive sports practice dissociates the disability label from a pathological view [33]. Moreover, adaptive sports may improve self-image and cognitive performance [14,31].

For the reasons stated above, the development of adaptive sports programs for people with physical disabilities can be considered an important and profitable health strategy in economic terms. Since this strategy requires a certain initial investment in sports equipment; adaptive sports could be a good strategy for people with physical disabilities to take a more active role in their health improvement processes and could also reduce the health costs generated by the complications associated with physical disability. In addition, improving social participation [12,32] and family life [29] through the practice of adaptive sports [29,30] could contribute to diversifying social resources, thus reducing the burden on the main caregiver and the associated healthcare costs.

### 4.1. Limitations

The interpretation of the results should be approached with caution, considering the limited number of primary studies that met the inclusion criteria in this meta-analysis. This fact can be attributed to various factors: (i) the literature has focused on therapeutic exercise or physical activity instead of adaptive sports practice, (ii) the challenges associated with conducting studies with a longitudinal design, and (iii) the limited availability of studies specifically targeting adults with physical disabilities. This limitation arises due to the inclusion of individuals with diverse forms of disabilities—physical, visual, or cognitive—in some studies. Nevertheless, international organizations distinguish physical disability as a distinct category from those mentioned earlier. The field of adaptive sports for adults with physical disabilities is still relatively unexplored, especially when focusing on the practice of sports with pre-established rules and no therapeutic objective but solely for sportive purposes. Conducting a meta-analysis of primary longitudinal studies involving participation in adaptive sports allows an examination of the impact on the quality of life for individuals with physical disabilities. If cross-sectional studies or therapeutic-based interventions were included, establishing a direct relationship between adaptive sports engagement and changes in quality of life would be less feasible. The present meta-analysis allows quantifying and showing the influence of adaptive sports on the quality of life of people with physical disabilities.

### 4.2. Future Research

This meta-analysis may be a foundational step toward understanding the impact of adaptive sports on the lives of individuals with physical disabilities. It would be highly interesting to continue exploring the practice of adaptive sports as a health tool. By doing so, this study could give insights into the influence that sports practice has on various aspects of the lives of individuals with and without disabilities who engage in sports, as well as its impact on different age groups or sports modalities practiced.

## 5. Conclusions

The practice of adaptive sports exhibited a positive influence on the mental quality of life of adults with physical disabilities. To our knowledge, this meta-analysis is the first that includes primary studies involving adults with physical disabilities who participate in adaptive sports, regardless of the underlying pathology. Adaptive sport could be considered a potential stand-alone public health intervention, providing new perspectives and motivating individuals with physical disabilities to actively engage in their own health process. Based on the obtained results, the promotion of adaptive sports should be considered at a policy level, encouraging greater investment and support as a health resource. However, further studies are needed to confirm the data obtained in this meta-analysis.

## Figures and Tables

**Table 1:** Summary of characteristics for included studies in the meta-analysis.

Author, Year	Design and QOL Tool	Sample Description and Objective	Intervention	Results and Conclusions
Ng et al., 2019 [14]	Quasi-experimental PROMIS-GH	EG: n = 6. Mean age: 49 yr. CG: n = 6. Mean age: 55 yr. Disability etiology: Multiple sclerosis. Objective: To examine whether ballroom dance could enhance participants’ quality of life.	EG: 6–8 weeks. Once a week, 1 h/session. Dances: Rumba, waltz, foxtrot, and push-pull. CG: No intervention.	Pre–post analysis: EG: Dance group reported improvements in health-related quality of life.
Barak et al., 2016 [15]	Pre–post study WHOQOL-BREF	IC: n = 9 NIC: n = 7 Recreational boccia: n = 14 Control: n = 13 Mean age: 46.46 yr. Disability etiologies: Cerebral palsy, multiple sclerosis, traumatic brain injury, and Friedreich ataxia. Objective: To assess the impact of a competitive boccia training program on the quality of life, in comparison to a recreational boccia training program and rehabilitation program, among residents of a rehabilitation center for individuals with severe chronic physical disabilities.	EG 1 and 2 (IC and NIC): Rehabilitation program, boccia training 3 times/week 1.5 h/session, and strength training program twice a week 1 h/session. EG 3 (Recreational boccia): Rehabilitation program and training tactics 2 sessions per week. CG: Rehabilitation program.	Pre–post analysis: EG: The three groups significantly improved in WHOQOL-BREF physical domain. CG: Significant improvements in WHOQOL-BREF physical and psychological domains.
Jackson et al., 2012 [24]	Single-group repeated- measures study MSQOL-54	n = 11. Disability etiology: Multiple sclerosis. Objective: To evaluate the impact of a kickboxing program on quality of life of participants.	Five-week kickboxing program. Three times/week, 1 h/session including warm-up and cool-down activities.	Base–pretest analysis (5 weeks after base): Missing data. Pre–post analysis (1 week after program): No significant differences nor physical health (*p* = 0.110) or mental health (*p* = 0.213).
Aidar et al., 2007 [13]	RCT SF-36	EG: n = 15. Mean age: 50.3 yr. CG: n = 13. Mean age: 52.5 yr. Disability etiology: ischemic stroke Objective: To investigate the effect of swimming on the quality of life of participants.	EG: 12 weeks. Twice a week, 45–60 min/session including: Warm-up out of the water, exercises in the water, and swimming. CG: No intervention.	Pre–post analysis: EG: Significant differences (*p* < 0.05) in physical and mental health. CG: No significant differences in either physical or mental health. Between-group analysis: Significant differences favorable to EG in functional capacity, physical and social aspects, pain, overall health status, vitality, and mental health.

QOL: quality of life; EG: experimental group; CG: control group; IC: independent competition; yr: years; NIC: no independent competition; RCT: randomized controlled trial.

## Data Availability

All data included in the review are available under request.

## Abbreviations

WHO	World Health Organization
PRISMA	Preferred Reporting Items for Systematic Reviews and Meta-Analyses
PICO	Population, Intervention, Comparison, Outcome
RCT’s	Randomized Controlled Trial
RevMan	Review Manager 5.4.1 Software
GRADE	Grading of Recommendations Assessment, Development and Evaluation
SMD	Standardized Mean Difference
CI	Confidence Interval

## References

[1] Rodríguez C.C. (2004). Sobre el concepto de discapacidad. Una revisión de las propuestas de la OMS. Rev. Electrónica Audiol..

[2] Úbeda-Colomer J., Monforte J., Devís-Devís J. (2019). Physical activity of university students with disabilities: Accomplishment of recommendations and differences by age, sex, disability and weight status. Public Health.

[3] Declerck L., Kaux J.-F., Vanderthommen M., Lejeune T., Stoquart G. (2019). The Effect of Adaptive Sports on Individuals with Acquired Neurological Disabilities and Its Role in Rehabilitation. Curr. Sports Med. Rep..

[4] World Health Organization (2020). Directrices de la OMS Sobre Actividad Física y Hábitos Sedentarios: De un vistazo [WHO Guidelines on Physical Activity and Sedentary Behaviour: At a Glance].

[5] Yazicioglu K., Yavuz F., Goktepe A.S., Tan A.K. (2012). Influence of adapted sports on quality of life and life satisfaction in sport participants and non-sport participants with physical disabilities. Disabil. Health J..

[6] Malm C., Jakobsson J., Isaksson A. (2019). Physical activity and sports—Real health benefits: A review with insight into the public health of Sweden. Sports.

[7] Bascón-Seda A., Ramírez-Macías G. (2020). Are E-sports a sport? The term ‘sport’ in checkmate. Movimento.

[8] Gámez-Calvo L., Gamonales J.M., Hernández-Beltrán V., Muñoz-Jiménez J. (2022). Estado actual del balonmano para personas con discapacidad. revisión sistemática. Actual state of the handball for people with cerebral palsy. Systematic review Estado atual do andebol para pessoas com deficiência. Revisão sistemática. E-Balonmano.com.

[9] Ley C. (2009). Acción Psicosocial a Través del Movimiento, Juego y Deporte en Contextos de Violencia y de Conflicto. Investigación Sobre la Adecuación Sociocultural de la ‘Terapia a Través del Deporte’ y Evaluación de un Programa con Mujeres en Guatemala. Ph.D. Thesis.

[10] Nowak P.F., Kuśnierz C., Bajkowski D. (2021). Quality of Life Determinants in Professional Athletes. Psychol. Res. Behav. Manag..

[11] Bognar G. (2005). The Concept of Quality of Life. Soc. Theory Pract..

[12] Ravenek K.E., Ravenek M.J., Hitzig S.L., Wolfe D.L. (2012). Assessing quality of life in relation to physical activity participation in persons with spinal cord injury: A systematic review. Disabil. Health J..

[13] Aidar F.J., Silva A.J., Reis V.M., Carneiro A., Cotta S.C. (2007). Estudio de la calidad de vida en el accidente vascular isquémico y su relación con la actividad física. Rev. Neurol..

[14] Ng A., Bunyan S., Suh J., Huenink P., Gregory T., Gambon S., Miller D. (2019). Ballroom dance for persons with multiple sclerosis: A pilot feasibility study. Disabil. Rehabil..

[15] Barak S., Mendoza-Laiz N., Fuentes M.T.G., Rubiera M., Huyzler Y. (2016). Psychosocial effects of competitive Boccia program in persons with severe chronic disability. J. Rehabil. Res. Dev..

[16] Page M.J., McKenzie J.E., Bossuyt P.M., Boutron I., Hoffmann T.C., Mulrow C.D., Shamseer L., Tetzlaff J.M., Akl E.A., Brennan S.E. (2021). The PRISMA 2020 statement: An updated guideline for reporting systematic reviews. Int. J. Surg..

[17] Ouzzani M., Hammady H., Fedorowicz Z., Elmagarmid A. (2016). Rayyan—A web and mobile app for systematic reviews. Syst. Rev..

[18] Higgins J.P.T., Thomas J., Chandler J., Cumpston M., Li T., Page M.J., Welch V.A. (2022). Cochrane Handbook for Systematic Reviews of Interventions Version 6.3.

[19] Guyatt G.H., Oxman A.D., Vist G.E., Kunz R., Falck-Ytter Y., Alonso-Coello P., Schünemann H.J. (2009). GRADE: An emerging consensus on rating quality of evidence and strength of recommendations. Chin. J. Evid. Based Med..

[20] Miki Y., Kanayama C., Nakashima S., Yamasaki M. (2012). Health-related quality of life in active persons with spinal cord injury. Jpn. J. Phys. Fit. Sports Med..

[21] Kennedy P., Taylor N., Hindson L. (2006). A pilot investigation of a psychosocial activity coursefor people with spinal cord injuries. Psychol. Health Med..

[22] Medola F.O., Busto R.M., Marçal Â.F., Junior A.A., Dourado A.C. (2011). Sports on quality of life of individuals with spinal cord injury: A case series. Rev. Bras. Med. Esporte.

[23] Kljajić D., Eminović F., Dopsaj M., Pavlović D., Arsić S., Otašević J. (2016). The Impact of Sports Activities on Quality of Life of Persons with A Spinal Cord Injury: VPLIV ŠPORTNIH AKTIVNOSTI NA KAKOVOST ŽIVLJENJA OSEB S POŠKODBO HRBTENJAČE. Zdr. Varst..

[24] Jackson K., Edginton-Bigelow K., Cooper C., Merriman H. (2012). A Group Kickboxing Program for Balance, Mobility, and Quality of Life in Individuals with Multiple Sclerosis. J. Neurol. Phys. Ther..

[25] Laferrier J.Z., Teodorski E., Sprunger N., Cooper R.A., Schmeler M. (2017). Investigation of the Impact of Sports, Exercise and Recreation (ser) Participation on Psychosocial Outcomes in a Population of Veterans with Disabilities Using the Sports Outcome Research Tool and Comprehensive Uniform Survey (Sportacus). A Longitudinal S. J. Nov. Physiother..

[26] Hutzler Y., Chacham-Guber A., Reiter S. (2013). Psychosocial effects of reverse-integrated basketball activity compared to separate and no physical activity in young people with physical disability. Res. Dev. Disabil..

[27] Laferrier J.Z., Teodorski E., Cooper R.A. (2015). Investigation of the Impact of Sports, Exercise, and Recreation Participation on Psychosocial Outcomes in a Population of Veterans with Disabilities. Am. J. Phys. Med. Rehabil..

[28] Halabchi F., Alizadeh Z., Sahraian M.A., Abolhasani M. (2017). Exercise prescription for patients with multiple sclerosis; potential benefits and practical recommendations. BMC Neurol..

[29] Groff D.G., Lundberg N.R., Zabriskie R.B. (2009). Influence of adapted sport on quality of life: Perceptions of athletes with cerebral palsy. Disabil. Rehabil..

[30] Klenk C., Albrecht J., Nagel S. (2019). Social participation of people with disabilities in organized community sport: A systematic review. Ger. J. Exerc. Sport Res..

[31] Diaz R., Miller E.K., Kraus E., Fredericson M. (2019). Impact of Adaptive Sports Participation on Quality of Life. Sports Med. Arthrosc. Rev..

[32] Alhumaid M.M., Said M.A. (2023). Increased physical activity, higher educational attainment, and the use of mobility aid are associated with self-esteem in people with physical disabilities. Front. Psychol..

[33] Gutiérrez-Sanmartín M., Caus-Pertegáz N. (2006). Análisis de los motivos para la participación en actividades físicas de personas con y sin discapacidad. (Analysis of participation incentives in physical activities among people with and without disabilities). RICYDE Rev. Int. Cienc. Deporte.

